# *Leishmania (L.) amazonensis* Lainson & Shaw, 1972: A cruel parasite causing a high-morbidity disease with severe physical deformities and, secondarily, even death

**DOI:** 10.1590/0037-8682-0393-2024

**Published:** 2025-06-02

**Authors:** Fernando Tobias Silveira

**Affiliations:** 1 Instituto Evandro Chagas (Secretaria de Vigilância em Saúde e Meio Ambiente, Ministério da Saúde), Ananindeua, PA, Brasil.; 2 Núcleo de Medicina Tropical, Universidade Federal do Pará, Belém, PA, Brasil.

More than 40 years have passed since we started working with protozoan parasites of the genus *Leishmania* and leishmaniasis in the Brazilian Amazon. We have aimed to improve our understanding of the pathogenesis of American cutaneous leishmaniasis (ACL) and offer the most appropriate approaches for treating patients with different clinical forms of the disease^1^. Now, we should reflect on the relationship established with these parasites, particularly *Leishmania (L.) amazonensis* Lainson & Shaw, 1972, a parasite associated with the most severe and incurable clinical form of ACL, anergic diffuse cutaneous leishmaniasis (ADCL)^2^ ^,^ ^3^.

When Lainson and Shaw described *L. (L.) amazonensis* in 1972, a profound gap existed in the etiology of ACL in Brazil, mainly regarding ADCL. Although ADCL was known, its specific causative agent was not. From studies comparing the parasite in wild mammals (mainly small rodents of the genera *Oryzomys* and *Proechimys*), the sandfly vector *Lutzomyia flaviscutellata*, and humans with ADCL, Lainson and Shaw concluded that it was a new *Leishmania* species^4^.

As a researcher in leishmaniasis, expressing opposing feelings among these protozoan parasites, such as attraction and frustration, is challenging after so much time dedicated to understanding the pathogenic mechanisms of these parasites to establish the best approach for treating patients with ACL. In a specific case of ACL caused by *L. (L.) amazonensis*, the clinical-immunopathological spectrum of the disease may be established. This method allows us to identify not only the three clinical forms caused by this parasite, localized cutaneous leishmaniasis (LCL), borderline disseminated cutaneous leishmaniasis (BDCL), and ADCL, but also the most appropriate therapeutic approach for each form. However, none is effective against ADCL. 

Based on this clinical-immunological diagnosis, most patients may be capable of mounting an efficient cellular immune response, mainly comprising CD8^+^ T-cells. This response controls the development of the infection and leads to LCL, with a limited number of infiltrated skin lesions (usually 1-3) that respond well to antimony therapy. However, a smaller proportion of patients have a reduced ability to control the infection, as they do not develop a CD8^+^ T-cell immune response as efficiently as patients with LCL. This susceptibility allows the spread of dozens of secondary skin lesions from the primary lesion with an infiltrated plaque appearance, leading to BDCL, which requires twice the antimony dosage of LCL for healing. The prognosis of infection worsens if the parasite manages to immobilize, almost completely, the dynamics of the cellular immune response in its favor, including CD4^+^ and CD8^+^ T-cells. This interference allows the parasite to control the macrophage and its defense mechanisms against infection, including the production of nitric oxide derived from L-arginine through the action of enzyme-induced nitric oxide synthase, making it its prisoner and perfect host^5^. However, this appears to be a crucial and irreversible step toward the anergy of the cellular immune response and the establishment of ADCL^6^. Additionally, it is important to consider that intraspecific (genetic) variations in *L. (L.) amazonensis* may contribute to resistance to conventional drugs, such as amphotericin B^7^, potentially influencing the development of ADCL.

Some molecular mechanisms that the parasite uses to restrict the cellular immune response in its favor are already known, emphasizing the crucial role of species-specific antigens of glycoconjugated molecules, such as lipophosphoglycans (GLP) and phosphatidylserine (PS)^8^. However, the combined action of these mechanisms leads to anergy of the cellular immune response that can rarely be reversed. For instance, among the six ADCL cases in which we used a chemo-immunotherapeutic regimen of pentamidine associated with BCG+Leishvacin^9^, only two showed therapeutic success and were cured of the disease^10^ ^,^ ^11^. However, after the discontinuation of the production and supply of Leishvacin by BioBrás (Biochemistry of Brazil), following the recommendation of the World Health Organization, the treatment regimen was discontinued because of the incomplete definition of some vaccine antigens.

**FIGURE 1:**
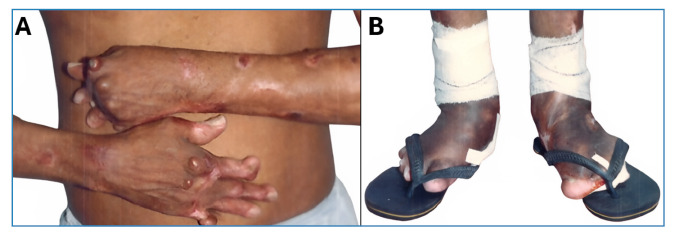
Anergic diffuse cutaneous leishmaniasis (ADCL) due to *L. (L.) amazonensis* in Pará State, Brazil, showing severe physical deformities due to osteomyelitis in the extremities, hands **(A)** and feet **(B)**.

Since then, no strategic advances have emerged to reverse the anergic state of the patients’ cellular immune response. Therefore, treatment has relied solely on chemotherapy with pentavalent antimonials, pentamidine, amphotericin B (conventional or liposomal), and miltefosine, all of which have shown limited effectiveness in controlling disease progression. However, a rare case of cure was observed in a patient who had been ill for over 30 years, following the isolated use of a nasal instillation of a single ampoule of meglumine antimoniate^12^, despite this approach having failed in four patients under our clinical supervision. Consequently, bone deformities that occur mainly in the extremities, feet, and hands (Figure 1), in which osteomyelitis causes progressive bone tissue loss, lead to limitations of various orders that negatively impact the socioeconomic lives of patients^13^ ^,^ ^14^. The consequences of the disease at this advanced stage can cause serious psycho-emotional disorders and even lead to suicide. This experience occurred with one (a man who had been sick for over 30 years) of the 19 patients with ADCL who have been monitored at the Leishmaniasis Outpatient Clinic of the Parasitology Department of the Evandro Chagas Institute, Secretary of Health and Environment Surveillance (Ministry of Health), Ananindeua, Pará State, Brazil. However, this experience has been reported by other professionals who treat patients with ADCL (Costa JML, personal communication).

Furthermore, we associated ADCL as an indirect cause of death in five other patients: four patients died because of renal failure resulting from a long treatment period (> 25 years) with potentially nephrotoxic drugs (mainly pentavalent antimony and pentamidine), and one patient developed malignant skin neoplasia (metastatic squamous cell carcinoma) associated with the carcinogenic process of chronic skin lesions. Most patients with ADCL (52.3%) acquired the disease at < 10 years of age. Of the 11 sick patients still alive, four are undergoing treatment for > 30 years (32-38 years), two have been followed up for 26 years, and five have been followed up for 12-15 years. After a series of 19 patients with ADCL, two were cured^10^ ^,^ ^11^.

Approximately 1.0 million cases of cutaneous leishmaniasis and another 50,000 cases of visceral leishmaniasis are recorded annually worldwide^15^. Therefore, the series presented here of only 19 cases of ADCL may not be relevant from a public health point of view. However, for all cases of cutaneous leishmaniasis and/or visceral leishmaniasis recorded annually worldwide, cure is expected with the drugs available for treatment. If a new ADCL case arrives at the outpatient clinic, we can predict that it will be another patient that we will treat for decades with little possibility of being cured. Therefore, *L. (L.) amazonensis* is a cruel parasite, and in the case of ADCL, scientific and humane perspectives should be considered.
